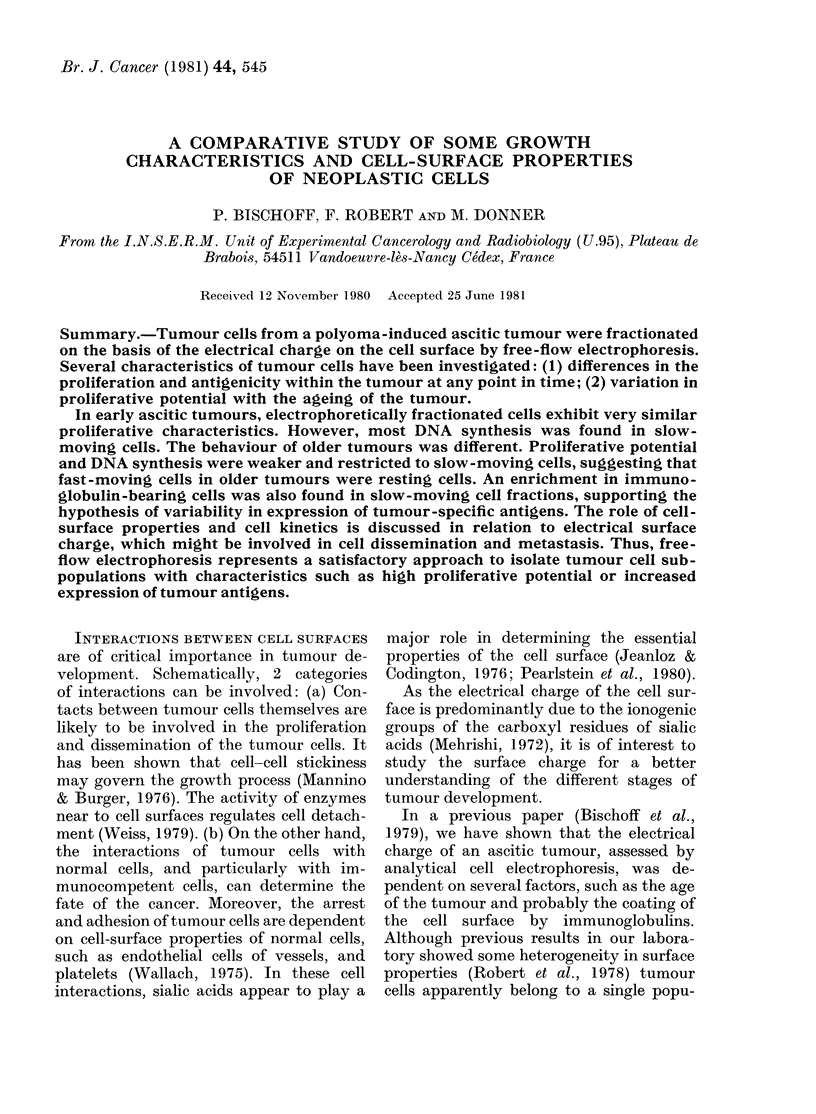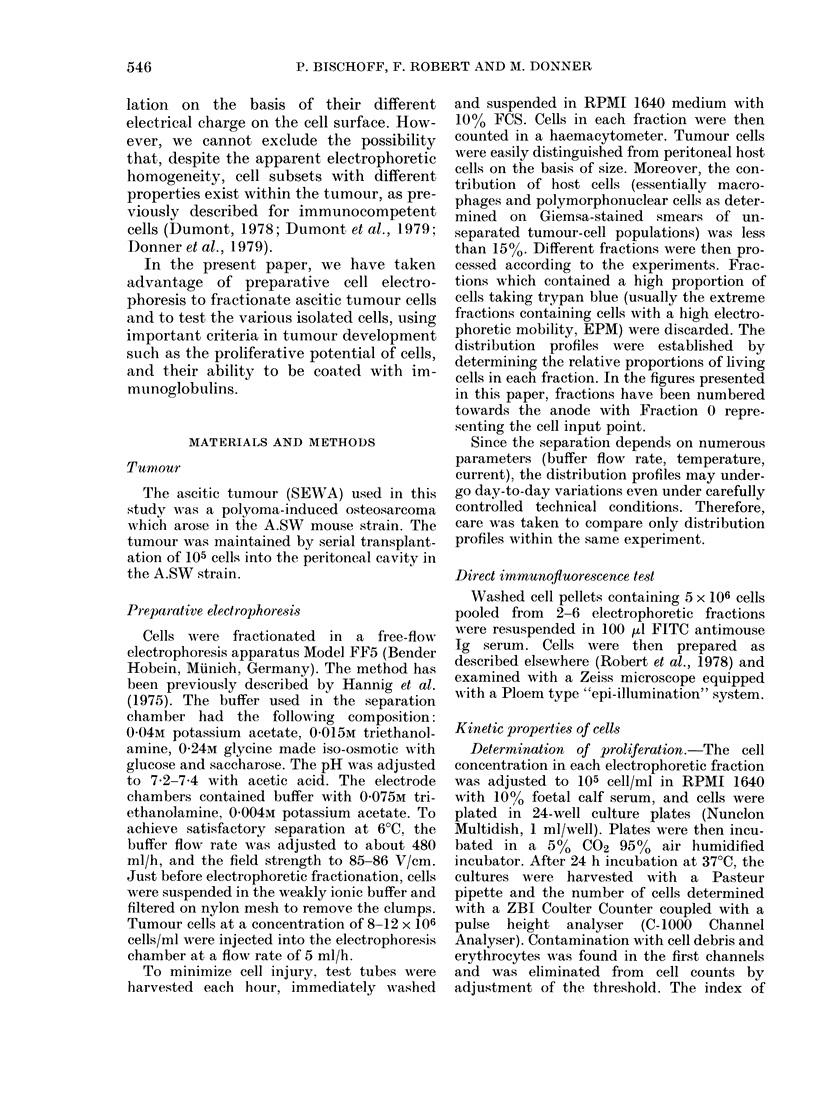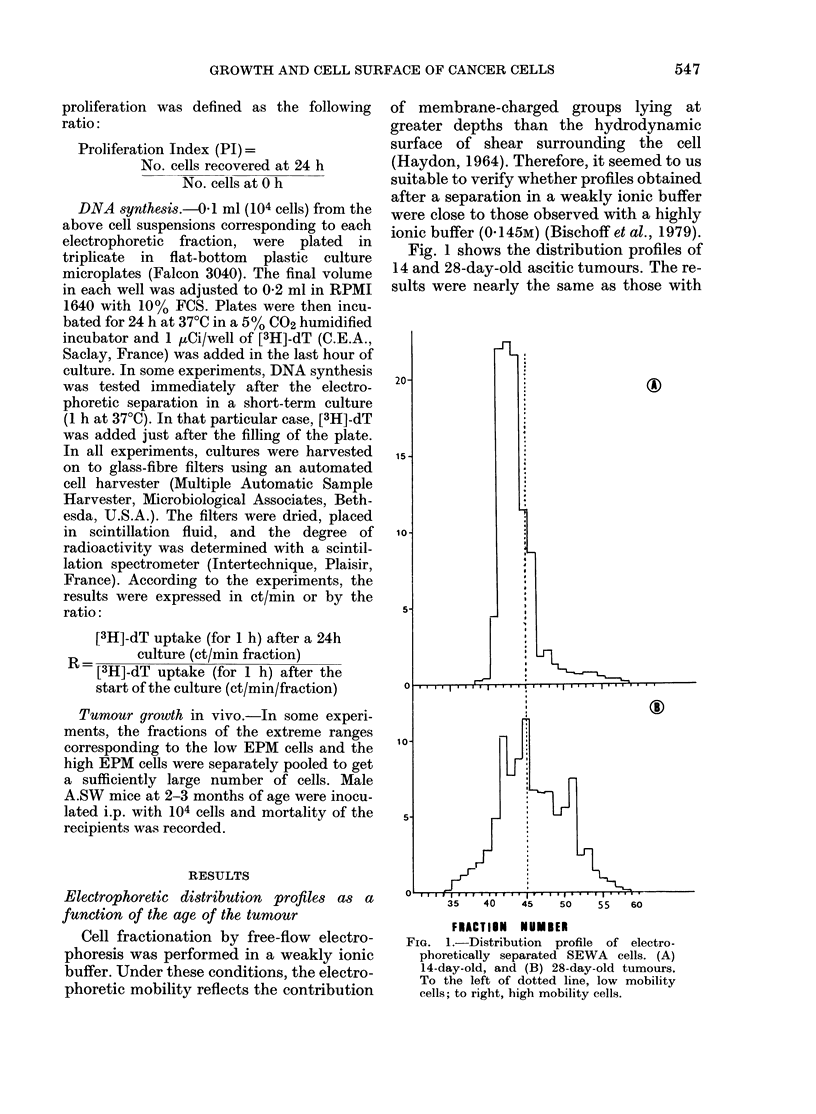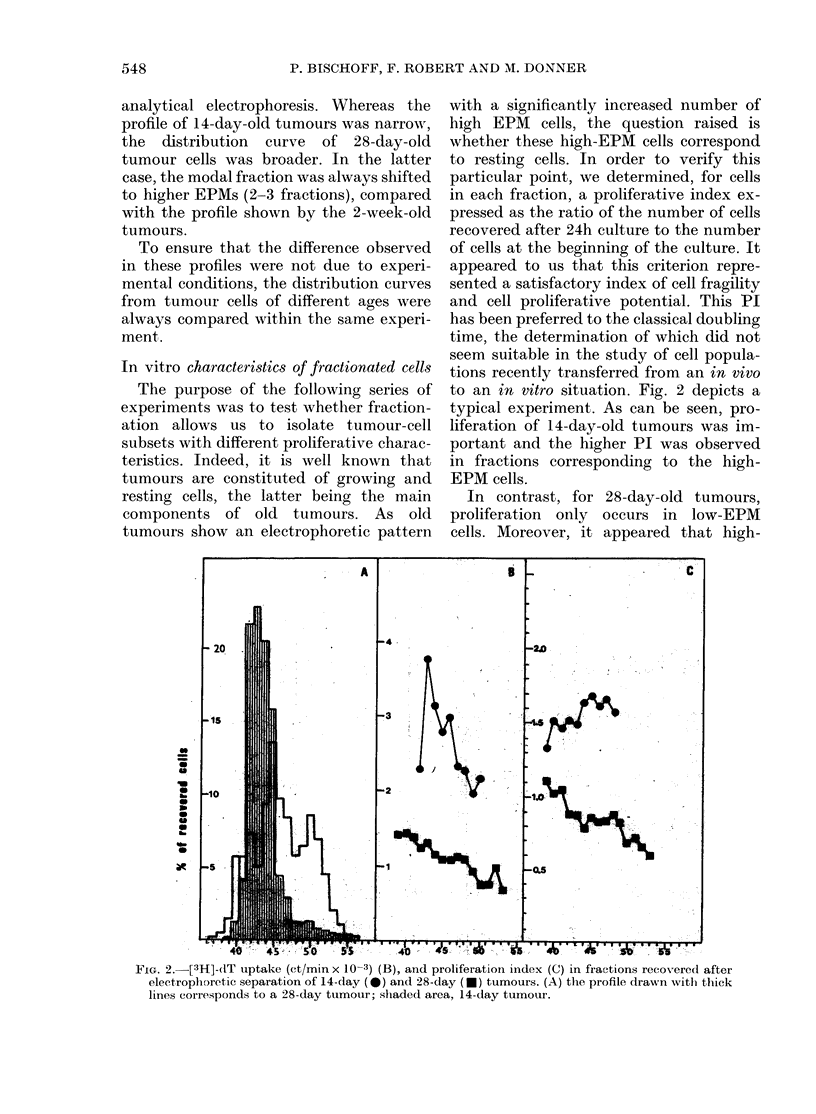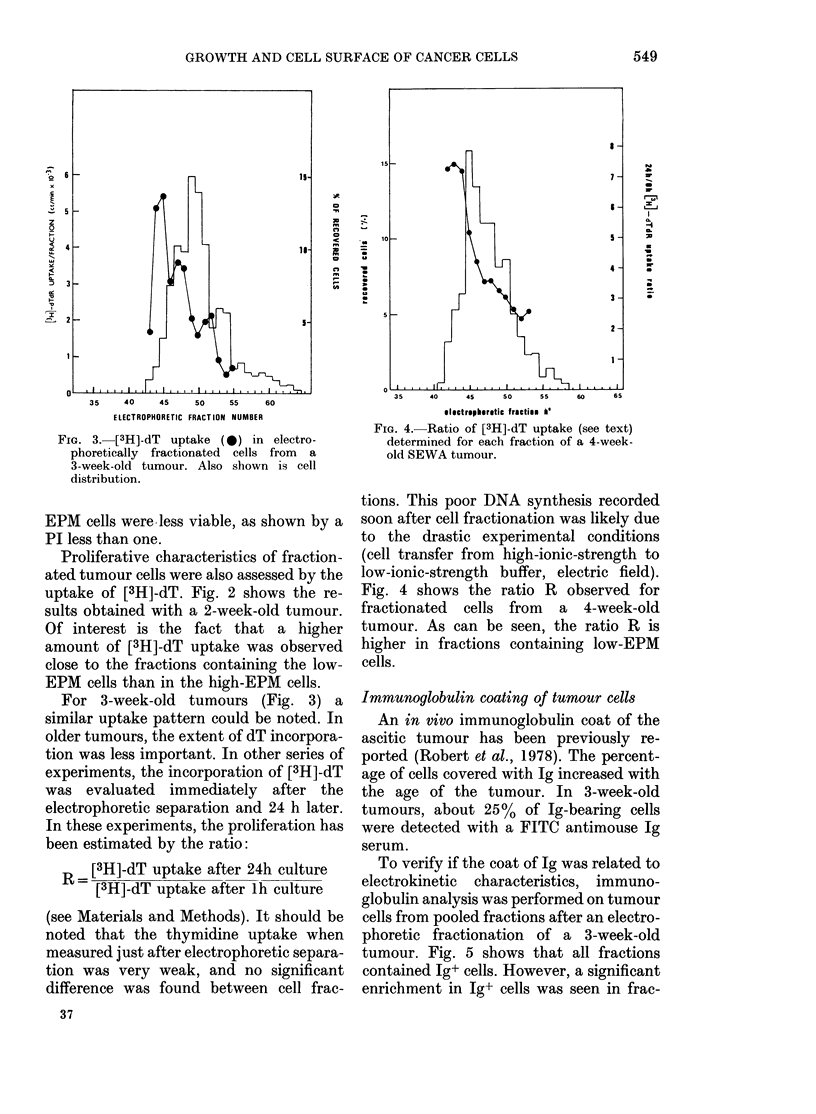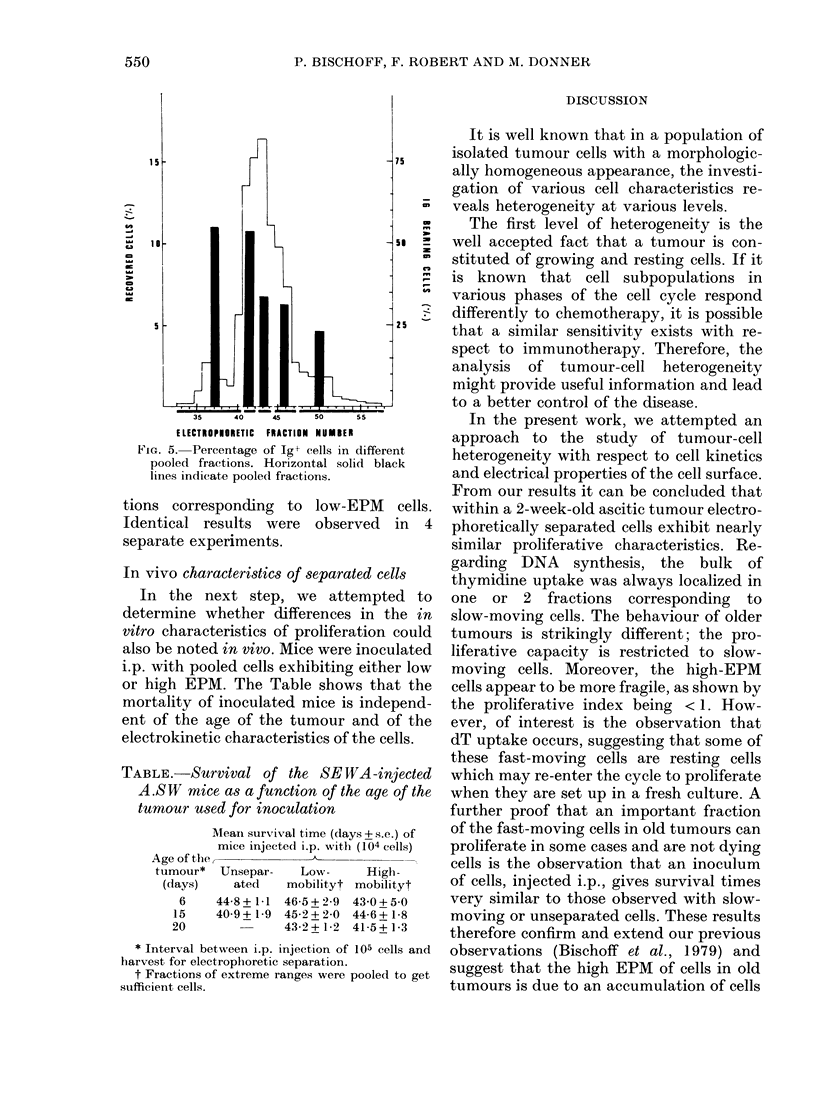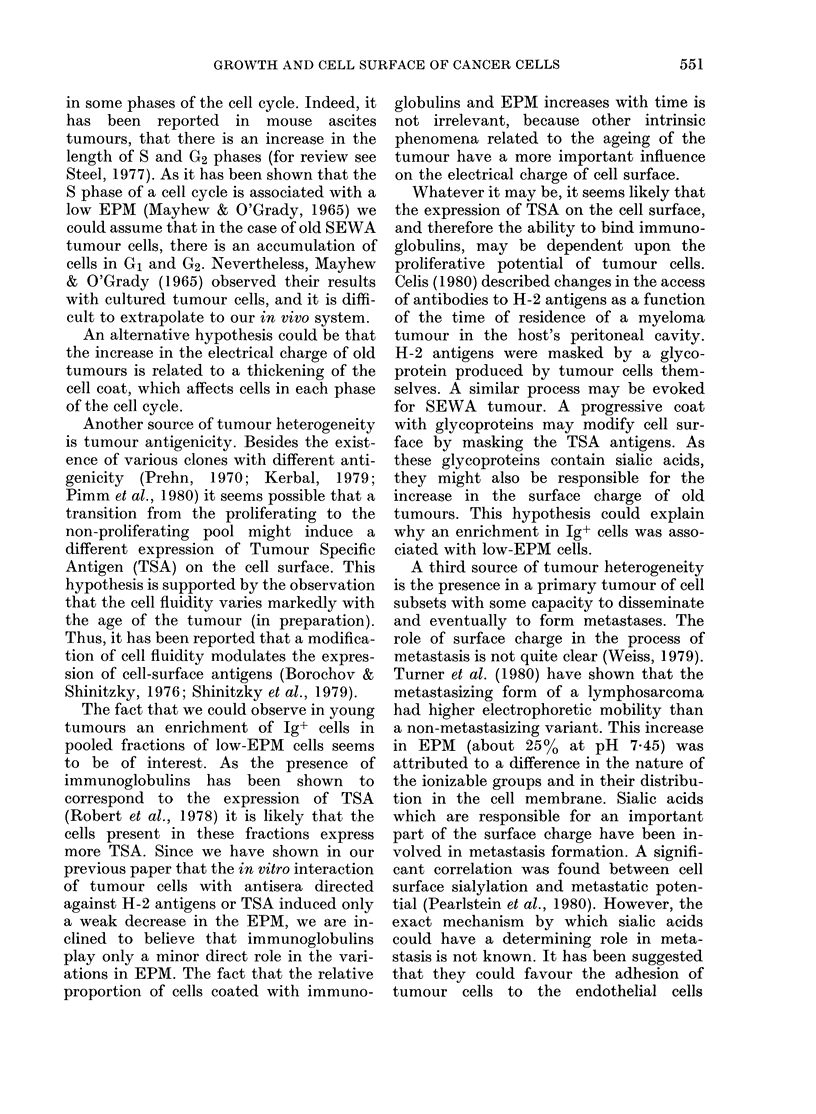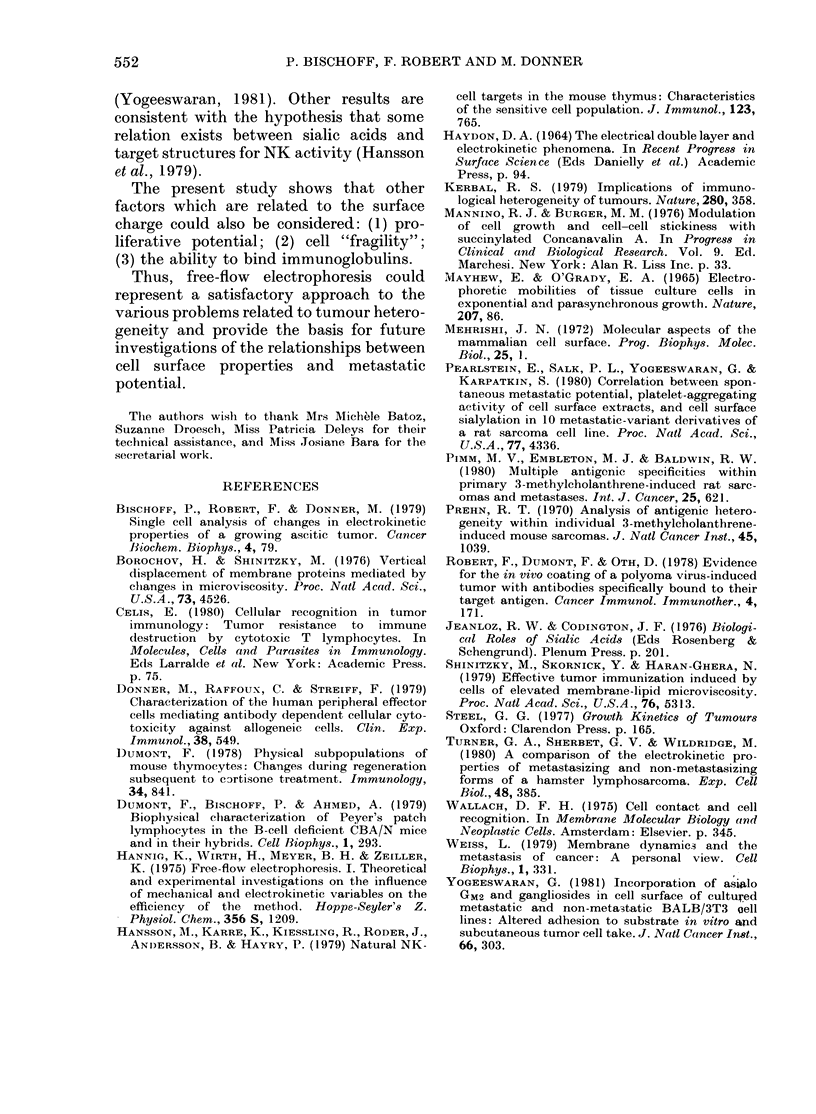# A comparative study of some growth characteristics and cell-surface properties of neoplastic cells.

**DOI:** 10.1038/bjc.1981.224

**Published:** 1981-10

**Authors:** P. Bischoff, F. Robert, M. Donner

## Abstract

Tumour cells from a polyoma-induced ascitic tumour were fractionated on the basis of the electrical charge on the cell surface by free-flow electrophoresis. Several characteristics of tumour cells have been investigated: (1) differences in the proliferation and antigenicity within the tumour at any point in time; (2) variation in proliferative potential with the ageing of the tumour. In early ascitic tumours, electrophoretically fractionated cells exhibit very similar proliferative characteristics. However, most DNA synthesis was found in slow-moving cells. The behaviour of older tumours was different. Proliferative potential and DNA synthesis were weaker and restricted to slow-moving cells, suggesting that fast-moving cells in older tumours were resting cells. An enrichment in immunoglobulin-bearing cells was also found in slow-moving cell fractions, supporting the hypothesis of variability in expression of tumour-specific antigens. The role of cell-surface properties and cell kinetics is discussed in relation to electrical surface charge, which might be involved in cell dissemination and metastasis. Thus, free-flow electrophoresis represents a satisfactory approach to isolate tumour cell subpopulations with characteristics such as high proliferative potential or increased expression of tumour antigens.


*Br. J. Cancer* (1981) **44**, 545

### A COMPARATIVE STUDY OF SOME GROWTH CHARACTERISTICS AND CELL-SURFACE PROPERTIES OF NEOPLASTIC CELLS

P. BISCHOFF, F. ROBERT AND M. DONNER

*From the I.N.S.E.R.M. Unit of Experimental Cancerology and Radiobiology (U.95), Plateau de Brabois, 54511 Vandoeuvre-lès-Nancy Cédex, France*

Received 12 November 1980 Accepted 25 June 1981

**Summary.—Tumour cells from a polyoma-induced ascitic tumour were fractionated on the basis of the electrical charge on the cell surface by free-flow electrophoresis. Several characteristics of tumour cells have been investigated: (1) differences in the proliferation and antigenicity within the tumour at any point in time; (2) variation in proliferative potential with the ageing of the tumour.**

**In early ascitic tumours, electrophoretically fractionated cells exhibit very similar proliferative characteristics. However, most DNA synthesis was found in slow-moving cells. The behaviour of older tumours was different. Proliferative potential and DNA synthesis were weaker and restricted to slow-moving cells, suggesting that fast-moving cells in older tumours were resting cells. An enrichment in immunoglobulin-bearing cells was also found in slow-moving cell fractions, supporting the hypothesis of variability in expression of tumour-specific antigens. The role of cell-surface properties and cell kinetics is discussed in relation to electrical surface charge, which might be involved in cell dissemination and metastasis. Thus, free-flow electrophoresis represents a satisfactory approach to isolate tumour cell sub-populations with characteristics such as high proliferative potential or increased expression of tumour antigens.**

INTERACTIONS BETWEEN CELL SURFACES are of critical importance in tumour development. Schematically, 2 categories of interactions can be involved: (a) Contacts between tumour cells themselves are likely to be involved in the proliferation and dissemination of the tumour cells. It has been shown that cell–cell stickiness may govern the growth process (Mannino & Burger, 1976). The activity of enzymes near to cell surfaces regulates cell detachment (Weiss, 1979). (b) On the other hand, the interactions of tumour cells with normal cells, and particularly with immunocompetent cells, can determine the fate of the cancer. Moreover, the arrest and adhesion of tumour cells are dependent on cell-surface properties of normal cells, such as endothelial cells of vessels, and platelets (Wallach, 1975). In these cell interactions, sialic acids appear to play a major role in determining the essential properties of the cell surface (Jeanloz & Codington, 1976; Pearlstein *et al.*, 1980).

As the electrical charge of the cell surface is predominantly due to the ionogenic groups of the carboxyl residues of sialic acids (Mehrishi, 1972), it is of interest to study the surface charge for a better understanding of the different stages of tumour development.

In a previous paper (Bischoff *et al.*, 1979), we have shown that the electrical charge of an ascitic tumour, assessed by analytical cell electrophoresis, was dependent on several factors, such as the age of the tumour and probably the coating of the cell surface by immunoglobulins. Although previous results in our laboratory showed some heterogeneity in surface properties (Robert *et al.*, 1978) tumour cells apparently belong to a single population on the basis of their different electrical charge on the cell surface. However, we cannot exclude the possibility that, despite the apparent electrophoretic homogeneity, cell subsets with different properties exist within the tumour, as previously described for immunocompetent cells (Dumont, 1978; Dumont *et al.*, 1979; Donner *et al.*, 1979).

In the present paper, we have taken advantage of preparative cell electrophoresis to fractionate ascitic tumour cells and to test the various isolated cells, using important criteria in tumour development such as the proliferative potential of cells, and their ability to be coated with immunoglobulins.

### MATERIALS AND METHODS

### *Tumour*

The ascitic tumour (SEWA) used in this study was a polyoma-induced osteosarcoma which arose in the A.SW mouse strain. The tumour was maintained by serial transplantation of $10^5$ cells into the peritoneal cavity in the A.SW strain.

### *Preparative electrophoresis*

Cells were fractionated in a free-flow electrophoresis apparatus Model FF5 (Bender Hobein, Münich, Germany). The method has been previously described by Hannig *et al.* (1975). The buffer used in the separation chamber had the following composition: 0·04M potassium acetate, 0·015M triethanolamine, 0·24M glycine made iso-osmotic with glucose and saccharose. The pH was adjusted to 7·2–7·4 with acetic acid. The electrode chambers contained buffer with 0·075M triethanolamine, 0·004M potassium acetate. To achieve satisfactory separation at 6°C, the buffer flow rate was adjusted to about 480 ml/h, and the field strength to 85–86 V/cm. Just before electrophoretic fractionation, cells were suspended in the weakly ionic buffer and filtered on nylon mesh to remove the clumps. Tumour cells at a concentration of $8–12 \times 10^6$ cells/ml were injected into the electrophoresis chamber at a flow rate of 5 ml/h.

To minimize cell injury, test tubes were harvested each hour, immediately washed and suspended in RPMI 1640 medium with 10% FCS. Cells in each fraction were then counted in a haemacytometer. Tumour cells were easily distinguished from peritoneal host cells on the basis of size. Moreover, the contribution of host cells (essentially macrophages and polymorphonuclear cells as determined on Giemsa-stained smears of unseparated tumour-cell populations) was less than 15%. Different fractions were then processed according to the experiments. Fractions which contained a high proportion of cells taking trypan blue (usually the extreme fractions containing cells with a high electrophoretic mobility, EPM) were discarded. The distribution profiles were established by determining the relative proportions of living cells in each fraction. In the figures presented in this paper, fractions have been numbered towards the anode with Fraction 0 representing the cell input point.

Since the separation depends on numerous parameters (buffer flow rate, temperature, current), the distribution profiles may undergo day-to-day variations even under carefully controlled technical conditions. Therefore, care was taken to compare only distribution profiles within the same experiment.

### *Direct immunofluorescence test*

Washed cell pellets containing $5 \times 10^6$ cells pooled from 2–6 electrophoretic fractions were resuspended in 100 $\mu$l FITC antimouse Ig serum. Cells were then prepared as described elsewhere (Robert *et al.*, 1978) and examined with a Zeiss microscope equipped with a Ploem type "epi-illumination" system.

### *Kinetic properties of cells*

*Determination of proliferation.*—The cell concentration in each electrophoretic fraction was adjusted to $10^5$ cell/ml in RPMI 1640 with 10% foetal calf serum, and cells were plated in 24-well culture plates (Nunclon Multidish, 1 ml/well). Plates were then incubated in a 5% $CO_2$ 95% air humidified incubator. After 24 h incubation at 37°C, the cultures were harvested with a Pasteur pipette and the number of cells determined with a ZBI Coulter Counter coupled with a pulse height analyser (C-1000 Channel Analyser). Contamination with cell debris and erythrocytes was found in the first channels and was eliminated from cell counts by adjustment of the threshold. The index of proliferation was defined as the following ratio:

$$\text{Proliferation Index (PI)} = \frac{\text{No. cells recovered at 24 h}}{\text{No. cells at 0 h}}$$

*DNA synthesis.*—0·1 ml ($10^4$ cells) from the above cell suspensions corresponding to each electrophoretic fraction, were plated in triplicate in flat-bottom plastic culture microplates (Falcon 3040). The final volume in each well was adjusted to 0·2 ml in RPMI 1640 with 10% FCS. Plates were then incubated for 24 h at 37°C in a 5% $CO_2$ humidified incubator and 1 $\mu$Ci/well of [$^3$H]-dT (C.E.A., Saclay, France) was added in the last hour of culture. In some experiments, DNA synthesis was tested immediately after the electrophoretic separation in a short-term culture (1 h at 37°C). In that particular case, [$^3$H]-dT was added just after the filling of the plate. In all experiments, cultures were harvested on to glass-fibre filters using an automated cell harvester (Multiple Automatic Sample Harvester, Microbiological Associates, Bethesda, U.S.A.). The filters were dried, placed in scintillation fluid, and the degree of radioactivity was determined with a scintillation spectrometer (Intertechnique, Plaisir, France). According to the experiments, the results were expressed in ct/min or by the ratio:

$$R = \frac{[^3\text{H}]\text{-dT uptake (for 1 h) after a 24h culture (ct/min fraction)}}{[^3\text{H}]\text{-dT uptake (for 1 h) after the start of the culture (ct/min/fraction)}}$$

*Tumour growth* in vivo.—In some experiments, the fractions of the extreme ranges corresponding to the low EPM cells and the high EPM cells were separately pooled to get a sufficiently large number of cells. Male A.SW mice at 2–3 months of age were inoculated i.p. with $10^4$ cells and mortality of the recipients was recorded.

### RESULTS

### *Electrophoretic distribution profiles as a function of the age of the tumour*

Cell fractionation by free-flow electrophoresis was performed in a weakly ionic buffer. Under these conditions, the electrophoretic mobility reflects the contribution of membrane-charged groups lying at greater depths than the hydrodynamic surface of shear surrounding the cell (Haydon, 1964). Therefore, it seemed to us suitable to verify whether profiles obtained after a separation in a weakly ionic buffer were close to those observed with a highly ionic buffer (0·145M) (Bischoff *et al.*, 1979).

Fig. 1 shows the distribution profiles of 14 and 28-day-old ascitic tumours. The results were nearly the same as those with

FIG. 1.—Distribution profile of electrophoretically separated SEWA cells. (A) 14-day-old, and (B) 28-day-old tumours. To the left of dotted line, low mobility cells; to right, high mobility cells.

analytical electrophoresis. Whereas the profile of 14-day-old tumours was narrow, the distribution curve of 28-day-old tumour cells was broader. In the latter case, the modal fraction was always shifted to higher EPMs (2–3 fractions), compared with the profile shown by the 2-week-old tumours.

To ensure that the difference observed in these profiles were not due to experimental conditions, the distribution curves from tumour cells of different ages were always compared within the same experiment.

### In vitro *characteristics of fractionated cells*

The purpose of the following series of experiments was to test whether fractionation allows us to isolate tumour-cell subsets with different proliferative characteristics. Indeed, it is well known that tumours are constituted of growing and resting cells, the latter being the main components of old tumours. As old tumours show an electrophoretic pattern with a significantly increased number of high EPM cells, the question raised is whether these high-EPM cells correspond to resting cells. In order to verify this particular point, we determined, for cells in each fraction, a proliferative index expressed as the ratio of the number of cells recovered after 24h culture to the number of cells at the beginning of the culture. It appeared to us that this criterion represented a satisfactory index of cell fragility and cell proliferative potential. This PI has been preferred to the classical doubling time, the determination of which did not seem suitable in the study of cell populations recently transferred from an *in vivo* to an *in vitro* situation. Fig. 2 depicts a typical experiment. As can be seen, proliferation of 14-day-old tumours was important and the higher PI was observed in fractions corresponding to the high-EPM cells.

In contrast, for 28-day-old tumours, proliferation only occurs in low-EPM cells. Moreover, it appeared that high-

FIG. 2.—[$^3$H]-dT uptake (ct/min × $10^{-3}$) (B), and proliferation index (C) in fractions recovered after electrophoretic separation of 14-day (●) and 28-day (■) tumours. (A) the profile drawn with thick lines corresponds to a 28-day tumour; shaded area, 14-day tumour.

FIG. 3.—[$^3$H]-dT uptake (●) in electrophoretically fractionated cells from a 3-week-old tumour. Also shown is cell distribution.

FIG. 4.—Ratio of [$^3$H]-dT uptake (see text) determined for each fraction of a 4-week-old SEWA tumour.

EPM cells were less viable, as shown by a PI less than one.

Proliferative characteristics of fractionated tumour cells were also assessed by the uptake of [$^3$H]-dT. Fig. 2 shows the results obtained with a 2-week-old tumour. Of interest is the fact that a higher amount of [$^3$H]-dT uptake was observed close to the fractions containing the low-EPM cells than in the high-EPM cells.

For 3-week-old tumours (Fig. 3) a similar uptake pattern could be noted. In older tumours, the extent of dT incorporation was less important. In other series of experiments, the incorporation of [$^3$H]-dT was evaluated immediately after the electrophoretic separation and 24 h later. In these experiments, the proliferation has been estimated by the ratio:

$$R = \frac{[^3\text{H}]\text{-dT uptake after 24h culture}}{[^3\text{H}]\text{-dT uptake after 1h culture}}$$

(see Materials and Methods). It should be noted that the thymidine uptake when measured just after electrophoretic separation was very weak, and no significant difference was found between cell fractions. This poor DNA synthesis recorded soon after cell fractionation was likely due to the drastic experimental conditions (cell transfer from high-ionic-strength to low-ionic-strength buffer, electric field). Fig. 4 shows the ratio R observed for fractionated cells from a 4-week-old tumour. As can be seen, the ratio R is higher in fractions containing low-EPM cells.

*Immunoglobulin coating of tumour cells*

An *in vivo* immunoglobulin coat of the ascitic tumour has been previously reported (Robert *et al.*, 1978). The percentage of cells covered with Ig increased with the age of the tumour. In 3-week-old tumours, about 25% of Ig-bearing cells were detected with a FITC antimouse Ig serum.

To verify if the coat of Ig was related to electrokinetic characteristics, immunoglobulin analysis was performed on tumour cells from pooled fractions after an electrophoretic fractionation of a 3-week-old tumour. Fig. 5 shows that all fractions contained $Ig^+$ cells. However, a significant enrichment in $Ig^+$ cells was seen in frac-

FIG. 5.—Percentage of Ig+ cells in different pooled fractions. Horizontal solid black lines indicate pooled fractions.

tions corresponding to low-EPM cells. Identical results were observed in 4 separate experiments.

### In vivo *characteristics of separated cells*

In the next step, we attempted to determine whether differences in the *in vitro* characteristics of proliferation could also be noted *in vivo*. Mice were inoculated i.p. with pooled cells exhibiting either low or high EPM. The Table shows that the mortality of inoculated mice is independent of the age of the tumour and of the electrokinetic characteristics of the cells.

TABLE.—*Survival of the SEWA-injected A.SW mice as a function of the age of the tumour used for inoculation*

| Age of the tumour* (days) | Mean survival time (days ± s.e.) of mice injected i.p. with ($10^4$ cells) | | |
|---|---|---|---|
| | Unseparated | Low-mobility† | High-mobility† |
| 6 | 44·8 ± 1·1 | 46·5 ± 2·9 | 43·0 ± 5·0 |
| 15 | 40·9 ± 1·9 | 45·2 ± 2·0 | 44·6 ± 1·8 |
| 20 | — | 43·2 ± 1·2 | 41·5 ± 1·3 |

* Interval between i.p. injection of $10^5$ cells and harvest for electrophoretic separation.

† Fractions of extreme ranges were pooled to get sufficient cells.

### DISCUSSION

It is well known that in a population of isolated tumour cells with a morphologically homogeneous appearance, the investigation of various cell characteristics reveals heterogeneity at various levels.

The first level of heterogeneity is the well accepted fact that a tumour is constituted of growing and resting cells. If it is known that cell subpopulations in various phases of the cell cycle respond differently to chemotherapy, it is possible that a similar sensitivity exists with respect to immunotherapy. Therefore, the analysis of tumour-cell heterogeneity might provide useful information and lead to a better control of the disease.

In the present work, we attempted an approach to the study of tumour-cell heterogeneity with respect to cell kinetics and electrical properties of the cell surface. From our results it can be concluded that within a 2-week-old ascitic tumour electrophoretically separated cells exhibit nearly similar proliferative characteristics. Regarding DNA synthesis, the bulk of thymidine uptake was always localized in one or 2 fractions corresponding to slow-moving cells. The behaviour of older tumours is strikingly different; the proliferative capacity is restricted to slow-moving cells. Moreover, the high-EPM cells appear to be more fragile, as shown by the proliferative index being < 1. However, of interest is the observation that dT uptake occurs, suggesting that some of these fast-moving cells are resting cells which may re-enter the cycle to proliferate when they are set up in a fresh culture. A further proof that an important fraction of the fast-moving cells in old tumours can proliferate in some cases and are not dying cells is the observation that an inoculum of cells, injected i.p., gives survival times very similar to those observed with slow-moving or unseparated cells. These results therefore confirm and extend our previous observations (Bischoff *et al.*, 1979) and suggest that the high EPM of cells in old tumours is due to an accumulation of cells in some phases of the cell cycle. Indeed, it has been reported in mouse ascites tumours, that there is an increase in the length of S and $G_2$ phases (for review see Steel, 1977). As it has been shown that the S phase of a cell cycle is associated with a low EPM (Mayhew & O'Grady, 1965) we could assume that in the case of old SEWA tumour cells, there is an accumulation of cells in $G_1$ and $G_2$. Nevertheless, Mayhew & O'Grady (1965) observed their results with cultured tumour cells, and it is difficult to extrapolate to our *in vivo* system.

An alternative hypothesis could be that the increase in the electrical charge of old tumours is related to a thickening of the cell coat, which affects cells in each phase of the cell cycle.

Another source of tumour heterogeneity is tumour antigenicity. Besides the existence of various clones with different antigenicity (Prehn, 1970; Kerbal, 1979; Pimm *et al.*, 1980) it seems possible that a transition from the proliferating to the non-proliferating pool might induce a different expression of Tumour Specific Antigen (TSA) on the cell surface. This hypothesis is supported by the observation that the cell fluidity varies markedly with the age of the tumour (in preparation). Thus, it has been reported that a modification of cell fluidity modulates the expression of cell-surface antigens (Borochov & Shinitzky, 1976; Shinitzky *et al.*, 1979).

The fact that we could observe in young tumours an enrichment of $Ig^+$ cells in pooled fractions of low-EPM cells seems to be of interest. As the presence of immunoglobulins has been shown to correspond to the expression of TSA (Robert *et al.*, 1978) it is likely that the cells present in these fractions express more TSA. Since we have shown in our previous paper that the *in vitro* interaction of tumour cells with antisera directed against H-2 antigens or TSA induced only a weak decrease in the EPM, we are inclined to believe that immunoglobulins play only a minor direct role in the variations in EPM. The fact that the relative proportion of cells coated with immunoglobulins and EPM increases with time is not irrelevant, because other intrinsic phenomena related to the ageing of the tumour have a more important influence on the electrical charge of cell surface.

Whatever it may be, it seems likely that the expression of TSA on the cell surface, and therefore the ability to bind immunoglobulins, may be dependent upon the proliferative potential of tumour cells. Celis (1980) described changes in the access of antibodies to H-2 antigens as a function of the time of residence of a myeloma tumour in the host's peritoneal cavity. H-2 antigens were masked by a glycoprotein produced by tumour cells themselves. A similar process may be evoked for SEWA tumour. A progressive coat with glycoproteins may modify cell surface by masking the TSA antigens. As these glycoproteins contain sialic acids, they might also be responsible for the increase in the surface charge of old tumours. This hypothesis could explain why an enrichment in $Ig^+$ cells was associated with low-EPM cells.

A third source of tumour heterogeneity is the presence in a primary tumour of cell subsets with some capacity to disseminate and eventually to form metastases. The role of surface charge in the process of metastasis is not quite clear (Weiss, 1979). Turner *et al.* (1980) have shown that the metastasizing form of a lymphosarcoma had higher electrophoretic mobility than a non-metastasizing variant. This increase in EPM (about 25% at pH 7·45) was attributed to a difference in the nature of the ionizable groups and in their distribution in the cell membrane. Sialic acids which are responsible for an important part of the surface charge have been involved in metastasis formation. A significant correlation was found between cell surface sialylation and metastatic potential (Pearlstein *et al.*, 1980). However, the exact mechanism by which sialic acids could have a determining role in metastasis is not known. It has been suggested that they could favour the adhesion of tumour cells to the endothelial cells

(Yogeeswaran, 1981). Other results are consistent with the hypothesis that some relation exists between sialic acids and target structures for NK activity (Hansson *et al.*, 1979).

The present study shows that other factors which are related to the surface charge could also be considered: (1) proliferative potential; (2) cell "fragility"; (3) the ability to bind immunoglobulins.

Thus, free-flow electrophoresis could represent a satisfactory approach to the various problems related to tumour heterogeneity and provide the basis for future investigations of the relationships between cell surface properties and metastatic potential.

The authors wish to thank Mrs Michèle Batoz, Suzanne Droesch, Miss Patricia Deleys for their technical assistance, and Miss Josiane Bara for the secretarial work.

### REFERENCES

BISCHOFF, P., ROBERT, F. & DONNER, M. (1979) Single cell analysis of changes in electrokinetic properties of a growing ascitic tumor. *Cancer Biochem. Biophys.*, **4**, 79.

BOROCHOV, H. & SHINITZKY, M. (1976) Vertical displacement of membrane proteins mediated by changes in microviscosity. *Proc. Natl Acad. Sci., U.S.A.*, **73**, 4526.

CELIS, E. (1980) Cellular recognition in tumor immunology: Tumor resistance to immune destruction by cytotoxic T lymphocytes. In *Molecules, Cells and Parasites in Immunology.* Eds Larralde *et al.* New York: Academic Press. p. 75.

DONNER, M., RAFFOUX, C. & STREIFF, F. (1979) Characterization of the human peripheral effector cells mediating antibody dependent cellular cytotoxicity against allogeneic cells. *Clin. Exp. Immunol.*, **38**, 549.

DUMONT, F. (1978) Physical subpopulations of mouse thymocytes: Changes during regeneration subsequent to cortisone treatment. *Immunology*, **34**, 841.

DUMONT, F., BISCHOFF, P. & AHMED, A. (1979) Biophysical characterization of Peyer's patch lymphocytes in the B-cell deficient CBA/N mice and in their hybrids. *Cell Biophys.*, **1**, 293.

HANNIG, K., WIRTH, H., MEYER, B. H. & ZEILLER, K. (1975) Free-flow electrophoresis. I. Theoretical and experimental investigations on the influence of mechanical and electrokinetic variables on the efficiency of the method. *Hoppe-Seyler's Z. Physiol. Chem.*, **356 S**, 1209.

HANSSON, M., KARRE, K., KIESSLING, R., RODER, J., ANDERSSON, B. & HAYRY, P. (1979) Natural NK-cell targets in the mouse thymus: Characteristics of the sensitive cell population. *J. Immunol.*, **123**, 765.

HAYDON, D. A. (1964) The electrical double layer and electrokinetic phenomena. In *Recent Progress in Surface Science* (Eds Danielly *et al.*) Academic Press, p. 94.

KERBAL, R. S. (1979) Implications of immunological heterogeneity of tumours. *Nature*, **280**, 358.

MANNINO, R. J. & BURGER, M. M. (1976) Modulation of cell growth and cell–cell stickiness with succinylated Concanavalin A. In *Progress in Clinical and Biological Research.* Vol. 9. Ed. Marchesi. New York: Alan R. Liss Inc. p. 33.

MAYHEW, E. & O'GRADY, E. A. (1965) Electrophoretic mobilities of tissue culture cells in exponential and parasynchronous growth. *Nature*, **207**, 86.

MEHRISHI, J. N. (1972) Molecular aspects of the mammalian cell surface. *Prog. Biophys. Molec. Biol.*, **25**, 1.

PEARLSTEIN, E., SALK, P. L., YOGEESWARAN, G. & KARPATKIN, S. (1980) Correlation between spontaneous metastatic potential, platelet-aggregating activity of cell surface extracts, and cell surface sialylation in 10 metastatic-variant derivatives of a rat sarcoma cell line. *Proc. Natl Acad. Sci., U.S.A.*, **77**, 4336.

PIMM, M. V., EMBLETON, M. J. & BALDWIN, R. W. (1980) Multiple antigenic specificities within primary 3-methylcholanthrene-induced rat sarcomas and metastases. *Int. J. Cancer*, **25**, 621.

PREHN, R. T. (1970) Analysis of antigenic heterogeneity within individual 3-methylcholanthrene-induced mouse sarcomas. *J. Natl Cancer Inst.*, **45**, 1039.

ROBERT, F., DUMONT, F. & OTH, D. (1978) Evidence for the *in vivo* coating of a polyoma virus-induced tumor with antibodies specifically bound to their target antigen. *Cancer Immunol. Immunother.*, **4**, 171.

JEANLOZ, R. W. & CODINGTON, J. F. (1976) *Biological Roles of Sialic Acids* (Eds Rosenberg & Schengrund). Plenum Press. p. 201.

SHINITZKY, M., SKORNICK, Y. & HARAN-GHERA, N. (1979) Effective tumor immunization induced by cells of elevated membrane-lipid microviscosity. *Proc. Natl Acad. Sci., U.S.A.*, **76**, 5313.

STEEL, G. G. (1977) *Growth Kinetics of Tumours* Oxford: Clarendon Press. p. 165.

TURNER, G. A., SHERBET, G. V. & WILDRIDGE, M. (1980) A comparison of the electrokinetic properties of metastasizing and non-metastasizing forms of a hamster lymphosarcoma. *Exp. Cell Biol.*, **48**, 385.

WALLACH, D. F. H. (1975) Cell contact and cell recognition. In *Membrane Molecular Biology and Neoplastic Cells.* Amsterdam: Elsevier. p. 345.

WEISS, L. (1979) Membrane dynamics and the metastasis of cancer: A personal view. *Cell Biophys.*, **1**, 331.

YOGEESWARAN, G. (1981) Incorporation of asialo $G_{M2}$ and gangliosides in cell surface of cultured metastatic and non-metastatic BALB/3T3 cell lines: Altered adhesion to substrate *in vitro* and subcutaneous tumor cell take. *J. Natl Cancer Inst.*, **66**, 303.